# Analytical review of 664 cases of penetrating buttock trauma

**DOI:** 10.1186/1749-7922-6-33

**Published:** 2011-10-13

**Authors:** Raimundas Lunevicius, Klaus-Martin Schulte

**Affiliations:** 1 Major Trauma Centre, King's College Hospital NHS Foundation Trust, King's Health Partners Academic Health Sciences Centre, Denmark Hill, London, SE5 9RS, UK

**Keywords:** buttock injury, penetrating trauma, shot wound, stab wound

## Abstract

A comprehensive review of data has not yet been provided as penetrating injury to the buttock is not a common condition accounting for 2-3% of all penetrating injuries. The aim of the study is to provide the as yet lacking analytical review of the literature on penetrating trauma to the buttock, with appraisal of characteristics, features, outcomes, and patterns of major injuries. Based on these results we will provide an algorithm. Using a set of terms we searched the databases Pub Med, EMBASE, Cochran, and CINAHL for articles published in English between 1970 and 2010. We analysed cumulative data from prospective and retrospective studies, and case reports. The literature search revealed 36 relevant articles containing data on 664 patients. There was no grade A evidence found. The injury population mostly consists of young males (95.4%) with a high proportion missile injury (75.9%). Bleeding was found to be the key problem which mostly occurs from internal injury and results in shock in 10%. Overall mortality is 2.9% with significant adverse impact of visceral or vascular injury and shock (*P *< 0.001). The major injury pattern significantly varies between shot and stab injury with small bowel, colon, or rectum injuries leading in shot wounds, whilst vascular injury leads in stab wounds (*P *< 0.01). Laparotomy was required in 26.9% of patients. Wound infection, sepsis or multiorgan failure, small bowel fistula, ileus, rebleeding, focal neurologic deficit, and urinary tract infection were the most common complications. Sharp differences in injury pattern endorse an algorithm for differential therapy of penetrating buttock trauma. In conclusion, penetrating buttock trauma should be regarded as a life-threatening injury with impact beyond the pelvis until proven otherwise.

## Background

The buttock comprises the lateral half of the lower most sagittal zone of the torso [1] where there is a particularly high density of vital structures above and below the peritoneum in the pelvis [2,3]. Sparse evidence points to the frequency of life-threatening visceral and vascular injuries in patients with penetrating trauma to the buttock [2,4,5]. Pelvic anatomy results in the possibility of major complications or death following penetrating buttock injury in any path of trajectory and in absence of hard vascular, abdominal, or pelvic signs [4].

A comprehensive review of data has not yet been provided as penetrating injury to the buttock is not a common condition accounting for 2-3% of all penetrating injuries [3,6-10]. Four previous reviews of the literature do however require additional research in terms of consistent patterns, peculiarities, and management [6-9].

The purpose of this study is to provide an analytical review of the literature on penetrating trauma to the buttock and to appraise the characteristics, features, outcomes, and patterns of major injuries. Recognition of specific patterns should enhance management of this trauma.

## Methods

The Entrez PubMed interface of MEDLINE database, EMBASE, Cochran, and CINAHL databases were searched using the following Medical Subject Heading (MeSH) keywords: "Injuries", "Wounds and Injuries", "Wound Penetrating"; each of these keywords was combined with the keyword "Buttocks". The term 'Penetrating Gluteal Injuries' was also used. This resulted in 1021 titles and abstracts of studies related to these terms which were then read on the basis of English language and relevance.

Commentaries and literature reviews were also taken into account. We excluded articles relating to blunt injury, acupuncture injury, intragluteal injection injury, needle stick accidents, iatrogenic injury of the gluteal arteries, wound closure, reconstructive surgery of gluteal defects, wound botulism, bone fracture complications, injury from ultraviolet light, burn injury, true aneurisms, malignancies, and animal studies.

Relevant studies on penetrating buttock injury in acute trauma setting were grouped and categorised chronologically. Clustered and individual data regarding the demographic characteristics, mechanism of injury, clinical mode of presentation, imaging, buttock zone wounded, injuries, management strategy, complications, and final outcome were accumulated from all the studies, either prospective or retrospective, and case reports. When calculations in main series were impossible due to the lack of particular data, they were performed through the use of informative subset with indication of the exact number of entered cases.

In order to assess outcomes of visceral, vascular, skeletal, nerve injuries as well as outcomes of major surgery after stabbing or shootings, the 95% confidence intervals of odds ratios were calculated. In order to detect differences in injury related with stabbing or shooting patterns and outcomes between two independent proportions a Z-test was chosen and employed as both sample sizes were greater than 30. The two-tailed test was used to assess the null hypothesis. Chi-square test with Yates' correction was employed to compare categorical "alive - dead" outcome. Two-tailed p values were calculated where by *P *< 0.05 was considered to indicate statistical significance. Microsoft Office XP Excel 2007 Worksheets were used for accumulation and analysis of data.

## Results

### Literature search

We identified four literature reviews [6-9], two prospective studies [11,12], twelve retrospective reviews [2-5,10,13-19], seventeen papers with case reports [6,8,20-33], and three commentaries [34-36]. 31 publication contributed patient data on a total of 664 patients. Although individual studies chosen for review had some variations in specific measures, they were conceptually similar. No articles reported population-based data on overall and type-specified buttock injury in relation to incidence and mortality. There were no systematic reviews or prospective randomised controlled trials identified. A summary of two prospective and twelve retrospective studies are shown in Table 1.

**Table 1:** Major endpoints of two prospective [11,12] and twelve retrospective reviews on penetrating buttock injury in acute trauma setting

Study/reference	Period years	Patients	Male	Mean age	Viscus/major vessel injury	Bony ring injury	Mean ISS	Major surgery*	Overall mortality	Morbidity in survivals	Concominant injuries	Hospital stay†	Cited articles	Contribution/concern
Velmahos et al.[11] (1997)	1	59	58	23	17 (29%)	5 (8%)	-	19(32.2%)	0	3 (15.8%)	High	7.2	11	Clinical examination is very accurate
Velmahos et al.[12] (1998)	1	10	-	-	-	-	-	-	0	-	-	-	14	Clinical examination is a reliable predictor
Maull et al. [13] (1979)	5	15	11	29	6 (54.5%)	-	-	12	0	5 (33%)	0	12	0	Liberal laparotomy advocated
Ivatury et al. [4] (1982)	4	60	57	-	16 (26.7%)	3 (5%)	-	16 (26.7%)	2 (3%)	14 (23%)	-	2 vs 18	3	Aggressive management
Vo et al. [5] (1983)	5	20	18	32	5 (25%)	2 (10%)	-	12 (60%)	0	5 (25%)	10 (50%)	-	2	Bullet's trajectory is important
Fallon et al. [14] (1988)	-	51	43	28.9	16 (31%)	0	-	25 (49%)	0	4 (8%)	High	-	4	Thorough evaluation and all investigations
Gilroy et al. [15] ( 1992)	6	8	7	33	8	-	-	8	2 (25%)	0	0	-	9	Danger of gluteal incision: vessels
Mercer et al. [3] (1992)	6	81	75	26	18 (22%)	4 (5%)	-	26% (21)	1 (1.2%)	-	-	-	6	Two zones of buttock: upper *vs *lower
Ferraro et al. [16] (1993)	2	70	68	25	34 (49%)	7 (17%)	11(1-45)	34 (49%)	3 (4%)	-	-	-	8	Sigmoidoscopy advocated
DiGiacomo et al. [2] (1994)	3	73	71	-	24 (33%)	10 (14%)	-	27 (37%)	1 (1.4%)	9 (12%)	-	-	10	Transpelvic bullet trajectory: surgery
Makrin et al. [17] (2001)	5	17	17	27	4 (23.5%)	0	-	2 (11.8%)	0	1 (6%)	0	4 (1-16)	5	Upper zone wounds carry higher risk
Susmallian et al. [18](2005)	5	39	38	-	4 (10.5%)	-	-	2 (5.1%)	0	0	0	-	6	Meticulous observation
Ceyran et al.[19] (2009)	17	27	27	-	-	0	-	25 (93%)	3 (11.1%)	1 (4.2%)	0	8 (7 -11)	7	Surgical approach and technique, if needed
Lesperance et.[10] (2009)	1.33	115	113	28	36 (31%)	40 (35%)	13 (1-75)	87 (76%)	7 (6%)	16 (14%)	66 (57%)	-	24	Military surgery experience
Summary	1 - 17	8 - 115	Most	Young	10.5 - 54.5%	0 - 35%	11 - 13	5.1 - 93%	0 - 25%	0 - 33%	High	Long	0 - 24	Dangerous injury/Contingencies possible

*Major surgery: laparotomy, suprapubic cystostomy, massive/operating room gluteal surgery (massive debridement included). †Hospital stay - mean/average. Values in parenthesis are percentages.

### Patient data

The analysis includes 664 patients for whom the minimal dataset was identified. Overall, 95.4% of cases (621/654) were males, and the median age was 29 (range 12-70). Missile injury accounted for 75.9% (504/664) and was mainly due to shooting (68.8%, 457/664), and rarely blasting (7.1%, 47 cases). Injury rate for stabbings was 23.8% (158/664). Impalement was rare with only 0.3% of cases (2/664). For 97 patients the zonal distribution was known, where by 66.0% (n = 64) were related to the upper zone of the buttock.

Clinical presentation on admission was known in 654 patients. 74 patients (11.3%) were regarded haemodynamically unstable and 56 (8.6%) were diagnosed to be in haemorrhagic shock. Peritoneal irritation was present in 48 (7.3%), gross rectal blood in 41 (6.3%), and gross haematuria in 27 (4.1%) patients. Massive external bleeding was documented in 15 patients, false aneurysm formation in 12, absence of distal pulse or cold painful leg in two, groin hematoma in two, and severe bone pain in three patients.

Initial diagnostic procedures were described by the authors as follows: diagnostic proctosigmoidoscopy in 295 (45.1%), angiography in 47 (7.2%), urology imaging (cystography, intravenous pyelography, urethrography) in 27 (4.1%) patients, and CT-scan for 10 (1.5%) patients. Retrograde irigoscopy and diagnostic peritoneal lavage were mentioned in a few reports.

### Treatment modalities

The treatment approaches were described in 654 patients. 176 (26.9%) patients underwent emergency laparotomy. 40 (6.1%) patients required extended gluteal surgery. The interventional radiology procedures were used as sole modality to control bleeding or target bullets in 12 patients (1.8%). 356 (54.4%) patients were observed without major procedure. Other surgical procedures such as debridement under general anaesthesia were performed in 16.5% (n = 108) of patients.

Laparotomy and extended gluteal surgery was performed for 207 patients in the subset of 615 patients with gunshot or stab trauma (33.7%). Laparotomy was performed on 12.0% of stabbed patients (19/158) and 32.4% (148/457) of patients that were shot (OR, 0.29; CI, 0.17-0.48; Z value 4.857; *P *< 0.001). Extended gluteal surgery was more often performed in the group of patients with stab injuries to the buttock: 33/158 (21.0%) operations in contrast to 7/457 (1.5%) operations in gunshot victims (OR, 16.97; CI, 7.33-39.29; Z value 8.32; *P *< 0.001).

### Outcomes

#### Mortality

Overall mortality rate was 2.9% (19/664). In terms of stabbing injury the mortality rate was 3.8% (6/158) and 2.6% (13/504) following missile injuries. Mortality rate due to gunshot injuries was 2.2% (10/457). 6.4% (3/47) of patients admitted for blast injuries had died. Both patients treated for impalement survived. Details related to each fatality due to penetrating injuries to the buttock are demonstrated in Table 2. Hypovolaemic shock, major surgical intervention, and visceral and/or vascular injury are all factors which have a significant impact on a lethal outcome (Table 3).

**Table 2:** Deaths due to penetrating injuries to the buttock in series of 664 cases

Author	Case no	Age	Gender	Injury Mechanism	Buttock or zone	Major finding on admission	Shock presentation	Bleeding	Surgical approach	Injuries	Surgical procedure	Cause of death
Ivatury [4]	1	15	Male	Stabbing	Left	Hypovolemic shock	ED	Internal	Laparotomy	IA	na	Shock
	2	26	Male	Stabbing	Left	Wound	Ward	Internal	Laparotomy	IA	Repair	Shock†
Gilroy [15]	3	45	Male	Shooting	Left	Hypovolemic shock	ED	External	Laparotomy	GA, bowels, bladder	Ligation, repair	Shock
	4	36	Male	Stabbing	Left	False aneurysm	Theatre	External	*Laparotomy	SGA	Ligation	Sepsis
Mercer [3]	5	17	Male	Shooting	Upper	Hypovolemic shock	ED	External & internal	Laparotomy	EIV	Repair	Shock
Ferraro [16]	6	na	na	Shooting	na	Hypovolemic shock	ED	na	Laparotomy	Pelvic veins	Pelvic packing	Shock
	7	na	na	Shooting	na	na	na	na	na	na	na	na
	8	na	na	Shooting	na	na	na	na	na	na	na	na
DiGiacomo [2]	9	na	na	Shooting	na	Hypovolemic shock	ED	Internal	Laparotomy	CIA, CIV Sigmoid colon	na	Shock
Ceyran [19]	10	na	Male	Stabbing	Left	Hypovolemic shock	ED	Internal	No surgery	IA	No	Shock
	11	na	Male	Stabbing	Right	Hypovolemic shock	ED	External	Gluteal	SGA	No	Shock†
	12	na	Male	Stabbing	Right	Hypovolemic shock	ED	External	Gluteal	SGA	No	Shock†
Lesperance [10]	13-16	na	na	Shooting	na	na	na	na	na	na	na	na
	17-19	na	na	Blast	na	na	na	na	na	na	na	na

IA - iliac artery, GA - gluteal artery, SGA - superior gluteal artery, EIV - external iliac vein, IIA - internal iliac artery, CIA - common iliac artery, CIV - common iliac vein, * Embolization was performed before laparotomy, † intraoperative deaths

**Table 3:** The impact of gender, injury mechanism, injury severity, and intervention on survival of patients with penetrating trauma to the buttock (n = 240)

Factor	Groups	Alive/Death	P*
Gender	male *vs *female	228/9 *vs *12/0	0.4917
Injury mechanism	stabbing *vs *shooting	64/5 *vs *176/4	0.1281
Hypovolemic shock	present vs not present	17/8 *vs *224/1	< 0.0001
Visceral/vascular injury	present *vs *not present	61/9 *vs *179/0	< 0.0001
Intervention extent	major *vs *minor/no surgery	89/9 *vs *151/0	0.0006

* Chi^2^-test with Yates' correction

#### Morbidity

The authors described 18 specific postoperative complications. As they did not adhere to a set of auditable complications, the following figures have mere descriptive value: wound infection (n = 16), sepsis or multiorgan failure (n = 10), small bowel fistula (n = 7 via laparotomy; n = 1 via gluteal wound), prolonged ileus or transient obstruction (n = 6), rebleeding (n = 5), local neurologic dysfunction or weakness of leg (n = 5), urinary tract infection (n = 4), myocardial infarction (n = 3), sacral decubitus (n = 3), stroke (n = 2), pleuropulmonary dysfunction (n = 2), thrombophlebitis/thrombosis (n = 2), and compartment syndrome of the lower extremity, perirectal hematoma, acute renal failure, paraplegia, malignant hypothermia, impotence (n = 1 for each complication). The seven most common complications constituted 75% of all complications (54 cases). 17 (2.6%) patients needed early postoperative reintervention.

### Patterns of major injuries

#### Pattern of major injuries related with penetrating trauma to the buttock

There were 615 cases of penetrating buttock injuries caused by stabbing or shooting after exclusion of blasting (n = 47) and impaled injuries (n = 2). There were 292 injuries to viscera, named vessels, bony pelvis, and nerves. Injuries of viscera (n = 173; 28.1%) prevail over injuries to major vessels (n = 81; 13.2%), bony pelvis (29 cases; 4.7%), or regional nerves (n = 9; 1.5%). Lumbosacral (n = 4) and sciatic nerve injuries (n = 5) were rare.

**Figure 1:**
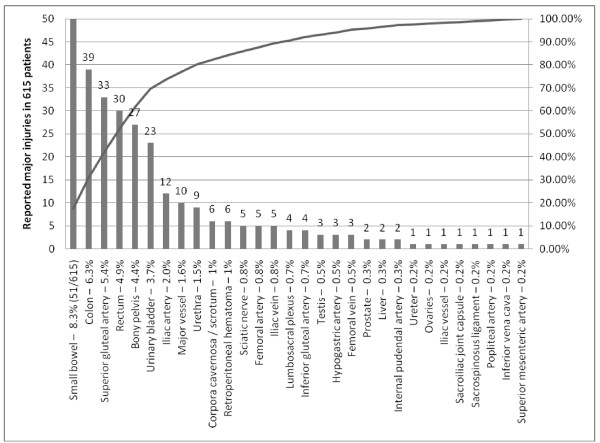
**Types of major injury in 615 patients with penetrating trauma to the buttock**.

The details of major injuries due to penetrating trauma to the buttock is shown in Figure 1. 30 anatomical terms were used to describe a particular injury type. The small bowel (8.3%), colon (6.3%), superior gluteal artery (5.4%), rectum (4.9%), bony pelvis (4.4%), bladder (3.7%), and iliac artery (2.0%) were on the top of the drawing scale of damaged anatomical structures. Summing up data on large bowel and major junctional vessel injury demonstrated that prevalence of injury to large bowel was 11.2% (n = 69); it was 2.9% for iliac artery or vein injury (n = 18), and 1.3% (n = 8) for femoral artery or vein injury. 10 major vessels injured due to penetrating buttock trauma were not named. Gluteal arteries were damaged in 37 patients (6.0%).

#### Pattern of major injuries related to stabbing

**Figure 2:**
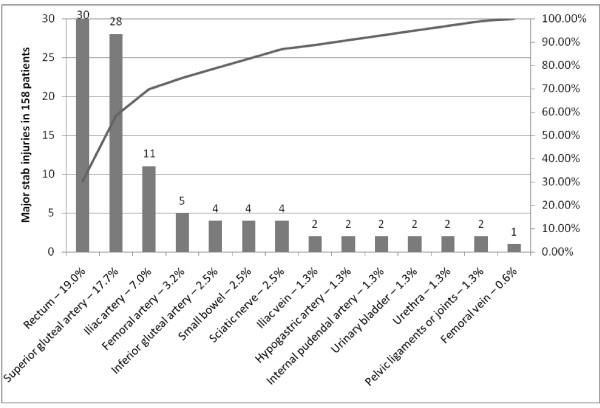
**Types of major injury related to stab trauma to the buttock in 158 patients**.

99 (63%) major injuries were identified in the subset of 158 patients with stab wounds (Figure 2). The prevalence of major vessel, visceral, sciatic nerve, and ligament/joint injury was 34.8% (n = 55), 24.1% (n = 38), 2.5% (n = 4), and 1.3% (n = 2), respectively. Rectum, superior gluteal artery, and iliac artery were the most frequently damaged major structures accounting for 19.0%, 17.7%, and 7.0%. In total, there were 32 injuries to gluteal arteries (20.3%), 13 injuries to iliac artery or vein (8.2%), and 6 injuries to femoral artery or vein (3.8%).

#### Pattern of major injuries related to shot wounds

**Figure 3:**
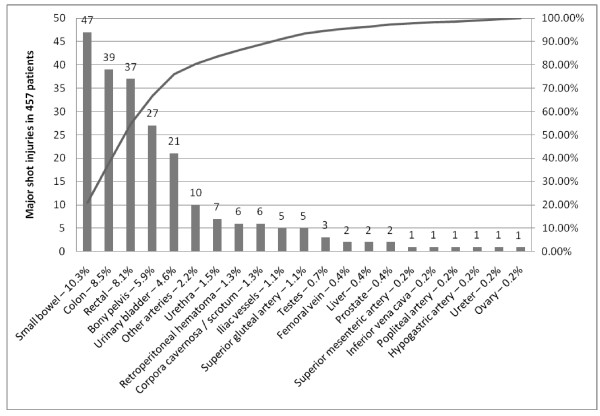
**Types of major injury related to shot trauma to the buttock in 457 patients**.

225 major injuries were identified in the subset of 457 patients with gunshot injury (Figure 3). There were 166 visceral injuries (36.3%), 27 injuries to the bony pelvis (5.9%), 26 injuries to major vessel (5.7%), 6 cases of retroperitoneal hematoma (1.3%), and 5 neurologic injuries (1.1%). The spectrum of major injuries associated with gunshot trauma to the buttock comprised 21 different types of injury. Injury of small bowel, colon, rectum, bony pelvis, and bladder were most frequent with 10.3%, 8.5%, 8.1%, 5.9%, and 4.6%, respectively. When colon and rectal injuries were collated, the prevalence of large bowel injury increased to 16.6% (n = 76).

### The pattern of major injury relating to injury mechanism

Table 4 demonstrates a higher frequency for all visceral and skeletal pelvic injuries in the patients with shot wounds. Injuries to the organs located more distally from the wound site (colon, small bowel, and bladder) were far more frequently damaged in patients with shot wounds to the buttock. Rectum and major vessels of the region (iliac vessels, femoral vessels, and gluteal arteries) were damaged more frequently in patients with stab wounds to the buttock.

**Table 4:** Stabbing *vs *shooting related major injuries of the buttock

Injuries	Stab wound n = 158	Shot wound n = 457	Odds Ratio	95% Confidence Internal	P*
Visceral:	38 (24%)	166 (36%)	0.56	0.37-0.84	0.006
Colon	0	39 (9%)	0.24	0.11-0.50	0.0003
Small bowel	4 (3%)	47 (10%)	0.23	0.08-0.64	0.004
Rectal	30 (19%)	37 (8%)	2.66	1.58-4.48	0.0003
Bladder	2 (1%)	21 (5%)	0.33	0.08-1.42	0.0097
Major vessel:	55 (35%)	26 (6%)	8.85	5.30-14.80	0.0001
Gluteal arteries:	32 (20%)	5 (1%)	22.96	8.76-60.14	0.0001
Superior gluteal artery	28 (18%)	5 (1%)	19.47	7.37-51.43	0.0001
Inferior gluteal artery	4 (3%)	0	49.97	5.28-473.4	0.005
Iliac vessels:	13 (8%)	5 (1%)	8.10	2.84-23.12	0.0001
Iliac artery	7 (4%)	1 (0.2%)	8.10	2.84-23.12	0.0003
Internal iliac artery	4 (3%)	0	49.97	5.28-473.4	0.0046
Femoral vessels:	6 (4%)	2 (0.4%)	8.98	1.79-44.96	0.005
Femoral artery	5 (3%)	0	50.30	6.72-376.39	0.001
Sciatic nerve	4 (3%)	1 (0.2%)	11.84	1.31-106.78	0.023
Bony pelvis	0	27 (6%)	0.25	0.10-0.59	0.004

Values in parenthesis are percentages. *Z test.

### Penetrating injuries to the upper *vs *lower zone of the buttock

A subset including 97 cases from two retrospective studies [3,17] and six case reports [21,22,25,27,29] provided data to assigns the main wound site to the upper or lower buttock region. Statistical results regarding penetrating injuries above and below the intertrochanteric line are shown in Table 5. There were 64 wounds to the upper zone (66.0%): 26 of them were related to stabbing and 38 to shooting. The lower zone of the buttock was targeted 33 times (34.0%): 15 subjects had stab wounds and 18 subjects had shot wounds. A prevalence of major injuries, either visceral/vascular, bony pelvis or sciatic nerve, was higher in patients with the entrance wound position above the intertrochanteric line. Visceral/vascular injuries were more frequent in patients with penetrating wounds in the upper zone of the buttock (25/64, 39.1% *vs *6/33, 18.2%; OR, 2.88; CI, 1.04-7.98; *P *< 0.05). The sensitivity of this test was 0.81, the positive predictive value was 0.39. Injury of soft tissue alone was more frequent in patients with penetrating injury to the lower zone of the buttock (32/64, 50.0% *vs *26/33, 78.8%; *P *< 0.05). The sensitivity of this test was 0.55, positive predictive value was 0.5.

**Table 5:** Penetrating injuries to the upper zone *vs *lower zone of the buttock

Injuries	Upper zone* n = 64	**Lower zone**†** n = 33**	Odds Ratio	95% Confidence Internal	**P**‡
Buttock soft tissue	32 (50%)	26 (79%)	0.27	0.10-0.71	0.012
SW	13 (50%)	10 (67%)	0.5	0.13-1.87	0.478
GSW	19 (50%)	16 (89%)	0.13	0.03-0.62	0.012
Visceral/Vascular/Bony	29 (45%)	6 (18%)	3.73	1.35-10.26	0.016
SW	11 (42%)	4 (27%)	2.02	5.51-8.05	0.506
GSW	18 (47%)	2 (11%)	7.2	1.45-35.73	0.019
Visceral/Vascular	25 (39%)	6 (18%)	2.88	1.04-7.98	0.063
SW	11 (42%)	4 (27%)	2.02	5.51-8.05	0.506
GSW	14 (37%)	2 (11%)	4.67	0.93-23.37	0.094
Bony pelvis	4 (6%)	0	4.78	0.58-39.10	0.353
SW	0	0	-	-	-
GSW	4 (11%)	0	4.90	0.58-41.69	0.383
Sciatic nerve	3 (5%)	1 (3%)	1.57	0.16-15.75	0.882
SW	2 (8%)	1 (7%)	1.17	0.10-14.06	0.616
GSW	1 (3%)	0	4.37	0.07-290.2	0.700

* 26 stab wounds, and 38 gunshot wounds, † 15 stab and 18 gunshot wounds. Values in parenthesis are percentages. ‡Z test . SW - stab wound, GSW - gunshot wound

## Discussion

It may be helpful to remind ourselves of the former surgical perspective on buttock trauma. Feigenberg (1992) reviewed four papers on stab wounds to the buttock and concluded that any stab wound to this body region should be regarded as potentially dangerous and every effort should be made to locate possible injuries [6]. Salim and Velmahos' review (2002) on abdominal gunshot wounds contains only one chapter regarding injury to the buttocks [7] and refers to one reference [11] pointing out that haemodynamically stable patients should be triaged (operation *vs *adjunct investigations) according to findings of physical examination. Aydin (2007) highlighted the importance of placing an acute false aneurysm in the differential diagnosis of an indurate, fluctuant, warm, erythematous posttraumatic gluteal mass [8]. The key statements of the review provided by Butt (2009) [9] are based on the summary of three papers [11,12,37] on gunshot wounds to the buttocks, back, and pelvis: firstly, the management of gunshot wounds of the buttocks should follow the same principles with anterior abdomen gunshot wounds; secondly, clinical examination is a reliable predictor for the need of an operation; thirdly, a rigid sigmoidoscopy is introduced per routine for all patients.

Case reports on penetrating buttock injury [6,8,19-33] highlight the importance of a thorough and aggressive evaluation of the patient [6], observation [23,27], prompt differential diagnosis [8,21,30,31], immediate assessment of the lower urinary tract [21,22], and lately the value of dynamic 2D and 3D CT-scanning and angiography [28]. They also highlight rare complications following high-velocity or low-velocity gunshot injury to the buttock where the bullet or pellet migrates to major veins such as inferior cava vein and hepatic veins [29] or if it reaches the right ventricle of the heart [23], needing a broad range of approaches ranging from open surgery to angioembolization [6,21,22], transjugular extraction of bullet from middle hepatic vein [29], image navigation surgery [33], gluteal surgery [28,32], laparoscopy [24], and laparotomy [6,20,21,25].

Our analytical review demonstrates that penetrating trauma to the buttock is a serious diagnostic and clinical concern with a mortality rate of 2.9%. Mortality of penetrating stab injuries to the buttock is comparable to that of extra-buttock regions of the body, such as penetrating injury to the posterior abdomen is 0-2% [37-39], the anterior abdomen 0-4.4% [40-43], the thoracoabdominal area 2.1% [44], and the chest 2.5-5.6% [44-46]. Mortality may be less in cohorts with isolated stab injury to the chest (1.46%) [45], or after exclusion of cardiac injuries (0.8%) [44]. Regarding pelvic or transpelvic gunshot trauma, mortality rates vary from 0-12.2% [11,47,48]. Cohorts with gunshot wounds to the limbs may show no mortality [49,50]. We conclude that penetrating injuries to the buttock poses a similar threat to the patient as penetrating trauma of any other body region.

Despite the fact that stab wound primarily cause loco-regional damage, whilst gunshot trauma is associated with frequent extraterritorial injury, stab wounds (3.8% mortality rate) are even more dangerous than missile wounds *per se *or gunshot wounds specifically (2.6% and 2.2% mortality rate, respectively). Injury of buttock due to impalement remains uncommon [26,51]. It is therefore recommended to classify impalement related injuries as a separate category of penetrating injuries [52].

Analysis of the associated major injuries due to penetrating trauma to the buttock reveals several unexpected particularities. The most commonly damaged particular organs and vessels were, in descending order, small bowel, colon, superior gluteal artery, and rectum. Injury of iliac artery and/or vein was a rare, but relevant finding with 2.9%. This counterintuitive finding is better understood on analysis of subgroups created according to injury mechanism.

**Figure 4:**
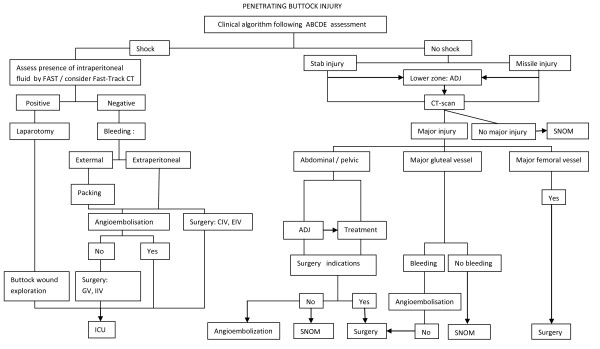
**Algorithm for management of penetrating trauma to the buttock**. FAST - Focused assessment with sonography for trauma. SNOM - Selective non-operative management. SE - Serial examination. ADJ - Adjuncts. Surgery indications: haemoperitoneum, injury of major or junctional vessel (CIV, EIV), perforation of bowel, peritonitis, not-stable bony pelvis, sciatic nerve transsection, necrotic/dirty soft tissue, urethra/ureter transsection, intraperitoneal bladder rupture (consider on individual basis). CIV - common iliac vessel. EIV - external iliac vessel. IIV - internal iliac vessel. ICU - Intensive care unit

As expected, stabbings were most frequently associated with injuries to gluteal arteries (20.3%), rectum (19.0%), and iliac vessels (8.2%). The prevalence of injuries to femoral artery or vein was 3.8%. Gunshot injuries frequently result in wider organ damage involving small bowel (10.3%), colon (8.5%), rectum (8.1%), bony pelvis (5.9%), and bladder injuries (4.6%). Table 4 provides ample evidence that gunshot and stab trauma of the buttock are actually two separate clinical entities. They require different diagnostic and surgical approaches which are summarised in Figure 4. In our view, such an approach based on empiric evidence might usefully supersede former algorithms by trying to address particular aspects of buttock trauma [2,5,14,17].

This review confirms the conclusion of two other authors [3,17] suggesting that injuries of upper zone of the buttock are associated with higher probability of viscus or major vessel injury comparing with injuries to the lower zone of the buttock. Table 5 reveals significant differentiation of injury patterns according to zone of primary injury site. However, the low positive predictive value does not recommend to rely on this criterion, for management strategies based on division of the buttock. On any account, the frequency of extraregional injury should prompt an aggressive and speedy computed tomography imaging approach to the entire abdomen and pelvis, complemented by a chest x-ray in all gunshot wounds to the buttock.

The current review contains a significant amount of historical data, bringing the use of endovascular approaches to only 1.8% in the current cohort. The advent of interventional radiological techniques should enable embolisation of pelvic vessels beside the level of the common or external iliac vessels [36,53].

Selective non-operative management of penetrating trauma to the buttock in stable patients without evidence of major organ injury is a successful approach [11]. Serial clinical examination should include per rectal examination, rigid sigmoidoscopy, and urinanalysis because of quite high probability of colorectal (11.2%) as well as bladder, urethra, and ureter injury (5.4%).

A classification of CT findings into three main groups of subset in relation to stable patients (abdominal/pelvis injury, gluteal vessel injury, and femoral vessel injury) is another feature of the algorithm (Figure 4). The rationale of this is the following: the buttocks should be regarded as a distinct anatomical/junctional zone in trauma surgery because patterns of penetrating injury and clinical characteristics as well as implications of buttock trauma disclosed in this paper correspond with general hallmarks of junctional trauma [54].

In terms of injury severity score, only Ferraro [16] and Lesperance [10] used the ISS scale. It is important to emphasise coding technique for penetrating buttock injury according to newest AIS 2005^©^Update 2008 [55]. It indicates that superficial (minor) penetrating injury to the buttock should be regarded as grade 1 (code 816011.1). When there is tissue loss >25 cm^2^, it should be regarded as grade 2 injury (code 816012.2), and when it is associated with blood loss >20% by volume, it has to be regarded as grade 3 injury (816013.3). Such injuries should be assigned to the external body region when calculating the ISS. However, if underlying anatomical structures are involved, documented diagnoses should be coded only, and they should be assigned to either the lower extremity body region or abdomen. Penetrating injuries involving a bone is coded as open fracture to the specific bone.

There are several limitations of this review. Publication bias, retrospective approach, clustered data, complexity of some injuries, and constrained nature of this study are the factors which undoubtedly cause our bias views. Prospective networked studies would be a better approach to the problem. The current review may help to design such studies.

In conclusion, penetrating buttock trauma should be regarded as a life-threatening injury with impact beyond the pelvis until proven otherwise.

## Competing interests

The authors declare that they have no competing interests.

## Authors' contributions

RL and KMS equally participated in the design of the study and interpretation of data. RL performed the literature review, statistical analysis of data, and drafting. KMS carried out the critical revision of the manuscript. Both authors read and approved the final manuscript.
